# Chromosomes of Asian Cyprinid Fishes: Genomic Differences in Conserved Karyotypes of ‘Poropuntiinae’ (Teleostei, Cyprinidae)

**DOI:** 10.3390/ani13081415

**Published:** 2023-04-20

**Authors:** Sudarat Khensuwan, Francisco de M. C. Sassi, Renata L. R. Moraes, Sitthisak Jantarat, Kriengkrai Seetapan, Krit Phintong, Weera Thongnetr, Sarawut Kaewsri, Sarun Jumrusthanasan, Weerayuth Supiwong, Petr Rab, Alongklod Tanomtong, Thomas Liehr, Marcelo B. Cioffi

**Affiliations:** 1 Department of Biology, Faculty of Science, Khon Kaen University, Muang, Khon Kaen 40002, Thailand; sudarat_k@kkumail.com (S.K.);; 2 Departamento de Genética e Evolução, Universidade Federal de São Carlos (UFSCar), Rodovia Washington Luiz Km. 235, C.P. 676, São Carlos 13565-905, Brazil; francisco.sassi@hotmail.com (F.d.M.C.S.); rlrdm@hotmail.com (R.L.R.M.); mbcioffi@ufscar.br (M.B.C.); 3 Department of Science, Faculty of Science and Technology, Prince of Songkla University, Pattani 94000, Thailand; sitthisak.j@psu.ac.th; 4 School of Agriculture and Natural Resources, University of Phayao, Tumbol Maeka, Muang, Phayao 56000, Thailand; kook82@hotmail.com; 5 Department of Fundamental Science, Faculty of Science and Technology, Surindra Rajabhat University, Muang, Surin 32000, Thailand; krit.p@srru.ac.th; 6 Division of Biology, Department of Science, Faculty of Science and Technology, Rajamangala University of Technology Krungthep, Bangkok 10120, Thailand; weera.t@mail.rmutk.ac.th; 7 Program in Biology, Faculty of Science, Buriram Rajabhat University, Muang, Buriram 31000, Thailand; sarawut.ks@bru.ac.th (S.K.); sarun.jum@bru.ac.th (S.J.); 8 Faculty of Applied Science and Engineering, Khon Kaen University, Nong Khai Campus, Muang, Nong Khai 43000, Thailand; weersu@kku.ac.th; 9 Laboratory of Fish Genetics, Institute of Animal Physiology and Genetics, Czech Academy of Sciences, Rumburská 89, 277 21 Liběchov, Czech Republic; rab@iapg.cas.cz; 10 Institute of Human Genetics, University Hospital Jena, 07747 Jena, Germany

**Keywords:** Ag-NOR, ribosomal DNA, repetitive DNAs, comparative genomic hybridization

## Abstract

**Simple Summary:**

All Poropuntiinae fish species are diploid and have 50 chromosomes in their cells; however, their karyotypes differ (the organization of chromosomes according to size and shape). The goal of this study is to compare the genomic differences between their conserved karyotypes using conventional and molecular cytogenetic methods. We found distinct patterns in the distribution of ribosomal DNA and microsatellites, indicating that, while their karyotypes are conserved, these fishes have species-specific patterns. Our comparative genomic hybridization experiment reveals that any of their repetitive DNA content matches, highlighting the differences between such species. This study adds to our understanding of chromosome evolution in Cyprinidae fishes, which include diploid, tetraploid, and hexaploid species.

**Abstract:**

The representatives of cyprinid lineage ‘Poropuntiinae’ with 16 recognized genera and around 100 species form a significant part of Southeast Asian ichthyofauna. Cytogenetics are valuable when studying fish evolution, especially the dynamics of repetitive DNAs, such as ribosomal DNAs (5S and 18S) and microsatellites, that can vary between species. Here, karyotypes of seven ‘poropuntiin’ species, namely *Cosmochilus harmandi*, *Cyclocheilichthys apogon*, *Hypsibarbus malcomi*, *H. wetmorei*, *Mystacoleucus chilopterus*, *M. ectypus*, and *Puntioplties proctozysron* occurring in Thailand were examined using conventional and molecular cytogenetic protocols. Variable numbers of uni- and bi-armed chromosomes indicated widespread chromosome rearrangements with a stable diploid chromosome number (2n) of 50. Examination with fluorescence in situ hybridization using major and minor ribosomal probes showed that *Cosmochilus harmandi*, *Cyclocheilichthys apogon*, and *Puntioplites proctozystron* all had one chromosomal pair with 5S rDNA sites. However, more than two sites were found in *Hypsibarbus malcolmi*, *H. wetmorei*, *Mystacoleucus chilopterus*, and *M. ectypus*. The number of chromosomes with 18S rDNA sites varied amongst their karyotypes from one to three; additionally, comparative genomic hybridization and microsatellite patterns varied among species. Our results reinforce the trend of chromosomal evolution in cyprinifom fishes, with major chromosomal rearrangements, while conserving their 2n.

## 1. Introduction

Carps and barbs from Africa and Eurasia are included in Cyprinidae *sensu stricto* (Ostariophysi, Cypriniformes) [1], the most biodiverse freshwater fish family with more than 3000 species [2,3]. The phyletic status of this family was debated for several years and currently 11 subfamilies are recognized: Labeoninae, Probarbinae, Torinae, Smiliogastrinae, Cyprininae, Acrossocheilinae, Spinibarbinae, Schizothoracinae, Schizopygopsinae, and Barbinae, as well as ‘Poropuntiinae’ [1]. Around 100 informally named ‘Poropuntiinae’ (*sensu* [1]) species, predominantly distributed in Southeast Asia (Burma, Thailand, Laos, Cambodia, Vietnam, Malaysia, and Indonesia), are thought to have diverged from cyprinid stem about 37.2 Ma ago [2,3]. They represent fish species with big importance in local artisanal fisheries in countries of their occurrence; some are also subjects of the ornamental fish trade (*Balantinocheilus melanopterus*, *Barbonymus schwanenfeldi*, *Sawbwa resplendens*). This group occupies a basal sister position to other Cyprinidae lineages, such as Cyprinini, Schizothoracinae, Spinibarbinae, Acrossocheilini, Schizopygopsinae and Barbinae [2]. Only 26 species from 14 of the 16 recognized genera of ‘Poropuntiinae’ have been cytogenetically investigated so far exhibiting all a remarkable conservation of 2n = 50 (Table 1); an ancestral trait for cypriniform taxa (e.g., [2,3,4,5,6,7,8,9]). All these available karyotype records relied on Giemsa-stained chromosomes (Table 1); hence, ‘Poropuntiinae’ members have not yet been subjected to molecular cytogenetic studies.

Genetic studies have played a crucial role in understanding the evolutionary history and diversity of fish species. Indeed, fish are excellent models for cytogenetic studies because they exhibit diverse karyotypes, including diploid and polyploid genomes, in addition to sex chromosomes, which offer unique insights into chromosome structure and behavior. An important technique for describing biodiversity is cytogenetics, the study of chromosomes and karyotypes [10]. To comprehend significant trends in chromosomal evolution across several vertebrate taxa, it is now common to integrate traditional and molecular methodologies (e.g., [11,12,13,14]). Polyploid (tri, tetra and hexaploid) species are commonly found in Cyprinidae [3], especially in Barbinae, Schizopygopsinae, Spinibarbinae, Schizothoracinae, Cyprininae, Probarbinae and Torinae. On other hand, Labeoninae, ‘Poropuntiinae’, Acrossocheilinae and most Smiliogastrinae are strictly composed by diploid species. Examining cytogenetic information from species with significant chromosomal rearrangements can help us understand their evolution and diversification [15]. Nonetheless, only research on diploid numbers and karyotype composition is now available for the ‘Poropuntiinae’ [16].

Molecular cytogenetics have emerged as an essential tool for describing evolutionary patterns, particularly in clades where species maintain a shared diploid number. The abundance and the chromosomal location of the repetitive DNA fraction change significantly between genomes of closely related species, and these variations are generally species-specific [17]. In this context, comparative genomic hybridization (CGH) and fluorescence in situ hybridization (FISH) mapping of repetitive DNAs were extensively used in teleost chromosomal research [18,19,20,21,22,23]. Ribosomal DNAs, for example, were frequently mapped in fish genomes and have been shown to occur mostly in separated chromosomes, though syntenic association has been reported in some species. Similarly, microsatellites are abundant in eukaryotic genomes, where they are either inserted in coding regions of structural genes or between other repetitive sequences [10]. By this way, the mapping of such sequences provides insights into intrachromosomal rearrangements and evolution of related species karyotypes. By identifying chromosomal markers associated with desirable traits and facilitating the production of genetically improved fish stocks, cytogenetic studies have also contributed to fish breeding and aquaculture.

**Table 1:** Review of available cytogenetic data for representatives of ‘Poropuntiinae’ species analyzed up to now. The species analyzed in this study are highlighted in red. Chromosomes were classified following their arm ratios in m = metacentric, sm = submetacentric, st = subtelocentric, and a = acrocentric, and their fundamental number (NF, i.e., number of arms) are also displayed [24]. Nucleolar organizer regions (NORs)/18S rDNA carrying pairs are highlighted.

Species	2n	NF	Karyotype	NORs/18S rDNA Pairs	Reference
*Amblyrhynchichthys truncatus*	50	78	16m + 12sm + 22a	-	[25]
*Barbonymus altus*	50	86	12m + 14sm + 10st + 14a	2	[26]
*Barbonymus gonionotus*	50	66	2m + 4sm + 10st + 34a	-	[27]
*Barbonymus gonionotus*	50	72	2m + 20sm + 4st + 24a	-	[28,29]
*Barbonymus gonionotus*	50	74	12m + 12sm + 4st + 22a	-	[30]
*Balantiocheilos melanopterus*	50	70	14m + 6sm + 10st + 20a	-	[31]
*Balantiocheilos melanopterus*	50	74	6m + 18sm + 16st + 10a	2	[32]
*Barbonymus schwanenfeldi*	50	76	6m + 6sm + 14st + 24a	-	[27]
*Barbonymus schwanenfeldii*	50	84	6m + 28sm/st + 16a	-	[33]
*Cosmochilus harmandi*	50	82	22m + 10sm + 10st + 8a	-	[34]
*Cosmochilus harmandi*	50	84	12m + 16sm + 6st + 16a	8	[26]
*Cosmochilus harmandi*	50	92	20m + 22sm + 4st + 4a	2, 17	Present work
*Cyclocheilichthys apogon*	50	70	12m + 8sm + 6st + 24a	-	[28]
*Cyclocheilichthys apogon*	50	76	18m + 8sm + 4st + 20a	-	[31]
*Cyclocheilichthys apogon*	50	86	10m + 16sm + 10st + 14a	6	[26]
*Cyclocheilichthys apogon*	50	74	14m + 30sm + 6a	11, 14, 20	Present work
*Cyclocheilichthys armatus*	50	94	12m + 18sm + 14st + 6a	3, 7	[35]
*Cyclocheilichthys repasson*	50	78	12m + 16sm + 6st + 16a	-	[36]
*Cyclocheilichthys repasson*	50	84	6m + 6sm + 22st + 16a	-	[27]
*Cyclocheilos enoplos*	50	90	10m + 30sm + 4st + 6a	two pairs (sm, a)	[37]
*Cyclocheilos enoplos*	50	72	14m + 8sm + 10st + 18a	-	[38]
*Cyclocheilos enoplos*	50	78	16m + 12sm + 6st + 16a	-	[31]
*Hypsibarbus lagleri*	50	74	4m + 20sm + 26a	-	[39]
*Hypsibarbus malcolmi*	50	64	10m + 4sm + 36a	-	[36]
*Hypsibarbus malcolmi*	50	62	8m + 4sm + 38a	1, 5	Present work
*Hypsibarbus vernayi*	50	58	6m + 2sm + 4st + 38a	-	[39]
*Hypsibarbus wetmorei*	50	70	12m + 8sm + 6st + 24a	-	[28]
*Hypsibarbus wetmorei*	50	74	12m + 12sm + 4st + 22a	2	[40]
*Hypsibarbus wetmorei*	50	74	12m + 12sm + 2st + 24a	-	[39]
*Hypsibarbus wetmorei*	50	82	10m + 14sm + 8st + 18a	6	[26]
*Hypsibarbus wetmorei*	50	78	14m + 14sm + 22a	2	Present work
*Mystacoleucus argenteus*	50	76	6m + 20sm + 2st + 22a	-	[25]
*Mystacoleucus chilopterus*	50	72	8m + 14sm + 4st + 24a	1	Present work
*Mystacoleucus ectypus*	50	72	10m + 12sm + 8st + 20a	7	Present work
*Mystacoleucus marginatus*	50	76	16m + 10sm + 24a	-	[41]
*Mystacoleucus marginatus*	50	68	14m + 4sm + 2st + 30a	-	[31]
*Poropuntius chonglingchungi*	50	80	12m + 18sm + 20a	-	[42]
*Poropuntius deauratus*	50	74	14m + 10sm + 26a	-	[34]
*Poropuntius laoensis*	50	74	14m + 10sm + 10st + 16a	-	[43]
*Poropuntius normani*	50	72	10m + 12sm + 28a	-	[36]
*Poropuntius sinensis*	50	82	10m + 22sm + 18a		[44]
*Puntioplties falcifer*	50	80	14m + 16sm + 2st + 18a	-	[36]
*Puntioplties falcifer*	50	92	16m + 10sm + 16st + 8a		[27]
*Puntioplties proctozysron*	50	76	20m + 6sm + 6st + 18a	-	[38]
*Puntioplties proctozysron*	50	76	16m + 10sm + 24a	-	[37]
*Puntioplties proctozysron*	50	82	6m + 14sm + 12st + 18a	2	[45]
*Puntioplties proctozysron*	50	90	18m + 22sm + 6st + 4a	12	Present work
*Scaphognathops bandanensis*	50	64	10m + 6sm + 34a	-	[36]
*Scaphognathops bandanensis*	50	66	10m + 6sm + 34a	2	[16]
*Sikukia gudgeri*	50	68	10m + 8sm + 4st + 28a	-	[34]

The present study examined the chromosomal diversity of the ‘poropuntiinae’ fishes/species from Thailand, namely *Cosmochilus harmandi*, *Cyclocheilichthys apogon*, *Hypsibarbus malcomi*, *H. wetmorei*, *Mystacoleucus chilopterus*, *M. ectypus*, and *Puntioplties proctozysron*, using conventional (Giemsa staining and Ag-NOR impregnation) and molecular (distribution of repetitive DNA sequences using FISH with respective probes and CGH) cytogenetic tools. Our findings support the idea that cyprinifom fishes have undergone significant chromosomal rearrangements while maintaining their 2n. Overall, the findings demonstrated that the evolution of this cyprinid lineage was significantly influenced by structural chromosomal rearrangements such as pericentric inversions.

## 2. Materials and Methods

### 2.1. Individuals, Mitotic Chromosome Preparation and Ag-NOR Banding

**Figure 1:**
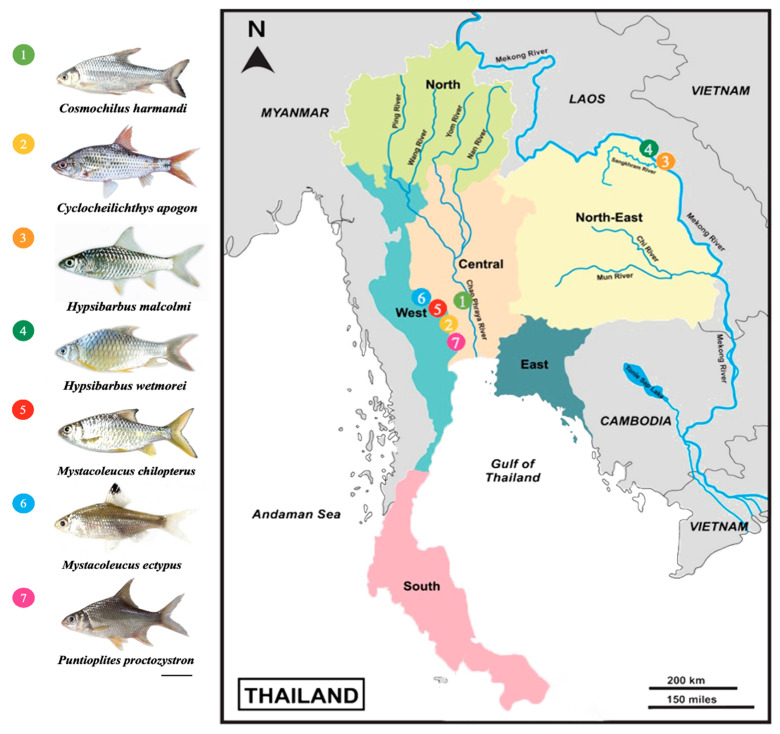
Thailand map showing the collection sites of the seven species studied including 1. *Cosmochilus harmandi* (light green circles); 2. *Cyclocheilichthys apogon* (yellow circles); 3. *Hypsibarbus malcolmi* (orange circles); 4. *Hypsibarbus wetmorei* (dark green circles); 5. *Mystacoleucus chilopterus* (red circles); 6. *Mystacoleucus ectypus* (blue circles); 7. *Puntioplites proctozystron* (pink circles). Scale bars = 1 cm (fish), 200 km (map) or 150 miles (map).

Individuals/fishes of seven representative ‘poropuntiins’ were collected from different natural ecosystems of wild regions in Thailand (Figure 1). Table 2 lists the numbers, sex, and locations of the individuals investigated. The specimens as vouchers were deposited in the fish collection of the Cytogenetic Laboratory, Department of Biology, Faculty of Science (KhonKaen University). All species analyzed here were properly identified using morphological criteria [46]. Mitotic chromosomes were obtained from the anterior kidney [47] and stained with 5% Giemsa. In brief, the animals were first injected in the abdomen with a 0.025% aqueous colchicine solution at a dose of 1 mL/100 g of weight. Then, specimens were euthanized after 50–60 min for the obtention of the rear kidney. Cells were dissociated with a sterile syringe in 5 mL of 0.075 M potassium chloride (KCl) and left for hypothonization at 37 °C for 25 min. Finally, turgid cells were fixed in Carnoy 2 (methanol 3:1 acetic acid) before being dropped into slides. The distribution of nucleolar organizer regions (Ag-NOR) was visualized according to the classical protocol, using silver nitrate (AgNO_3_) [48]. The fishes were collected with the authorization of the Animal Ethics Committee of KhonKaen University based on the Ethics of Animal Experimentation of the National Research Council of Thailand (Record No. IACUC-KKU-105/63).

### 2.2. Fluorescence In Situ Hybridization (FISH)

FISH experiments were performed under high stringency conditions [49] to identify both classes of ribosomal DNA (5S and 18S) and microsatellites (CA)_15_, (GC)_15_, (TA)_15_, and (CGG)_10_ sequences. The first ribosomal probe contained a 5S rDNA repeat copy and included 120 base pairs (bp) of the 5S rRNA transcribing gene in addition to 200 bp of the non-transcribed spacer (NTS) [50]. The second one corresponded to the 1400 bp segment of the 18S rRNA gene obtained via PCR from the nuclear DNA of the wolf fish *Hoplias malabaricus* [51]. Both probes were directly labeled with the Nick-Translation mix kit (Jena Bioscience, Jena, Germany), where 5S rDNA was labeled in red with Atto550-dUTP and the 18S rDNA was labeled in green with Atto448-dUTP, according to the manufacturer’s instructions. The microsatellite sequences were directly labeled with Cy-3 during the synthesis, as described by [52]. Slides were aged at 60 °C for 1h before being treated with RNAse solution (1.5 µL RNase A (10 mg/mL) in 1.5 mL 2 × SSC) at 37 °C also for 1 h. Chromosomes were denatured in 70% Formamide/2 × SSC solution at 72 °C for 3.15 min, whereas probes at 85 °C for 10 min then cooled at 4 °C before the application onto the slides. Hybridization occurred in a dark moist chamber overnight and then ended by a 1 × SSC wash at 65 °C for 5 min, followed by a 5 min wash with 4 × SSC/Tween and 1 min with 1 × PBS. Chromosomes were counterstained with DAPI diluted in Vectashield (Vector Laboratories, Burlingame, CA, USA).

### 2.3. Comparative Genomic Hybridization (CGH)

As substantial variation in karyotype structures was observed among ‘poropuntiin’ species, we selected those from distinct clades that exhibit different karyotype compositions to be compared. Total genomic DNA (gDNAs) from the males of *C. harmandi* and *M. chilopterus* was extracted from liver tissue using a purification DNA/RNA standard kit (Cellco Biotech, São Carlos, Brazil). The gDNA of *C. harmandi* and *M. chilopterus* were compared with that of *M. chilopterus* on metaphase chromosomes. For this purpose, gDNAs of *C. harmandi* and *M. chilopterus* were, respectively, directly labeled with Atto488-dUTP (green) and Atto550-dUTP (red) using the Nick-translation Labeling Kit (Jena Bioscience, Jena, Germany) at 15 °C for 3 h. To block common genomic repetitive sequences, we used unlabeled *C*_0_*t*-1 DNA (i.e., a subset of genomic DNA from each species that is enriched for highly and moderately repetitive sequences), prepared according to [53]. The final hybridization mixture for each experiment was composed of 500 ng labeled DNA of each compared species, plus 15 μg of male-derived *C*_0_*t*-1 DNA from the respective species and the hybridization buffer (50% formamide, 2 × SSC, 10% SDSC 10% dextran sulfate and Denhardt’s solution, pH 7.0). The CGH experiments were performed according to previous reports in related fish groups [18]. The probe mix with *C*_0_*t*-1 was denatured at 86 °C for 8 min, cooled at 4 °C and prehybridized at 37 °C for 1h. Hybridization occurred for 48h at 37 °C in a dark moist chamber. Post-hybridization washes were performed two times for 5 min in 1 × SSC at 65 °C, then in 4 × SSC/Tween at room temperature for 5min, following a short wash in 1 × PBS for 1 min. Slides were dehydrated in ethanol series (70%, 85%, 100%) for 2 min each before the application of DAPI solution as mentioned above in the FISH experiment.

### 2.4. Karyotyping and Image Processing

To confirm the 2n, karyotype structure, and FISH results, at least 20 metaphase spreads were analyzed per individual. Images were captured with an Axioplan II microscope (Carl Zeiss Jena GmbH, Jena, Germany) with CoolSNAP, and processed using Image-Pro Plus 4.1 software (Media Cybernetics, Silver Spring, MD, USA). Chromosomes were classified according to their arm ratios as metacentric (m), submetacentric (sm), subtelocentric (st), and acrocentric (a) [24].

## 3. Results

### 3.1. Karyotypes and Ag-NOR Phenotypes

**Figure 2:**
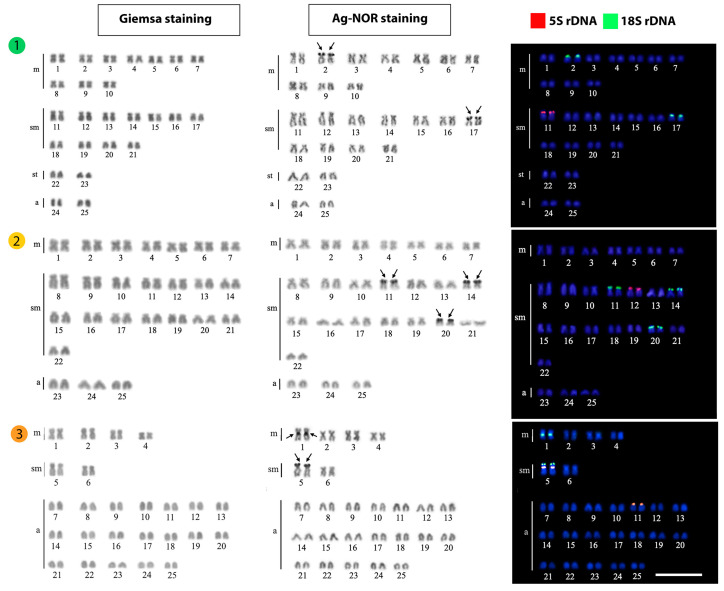
Karyotypes of ‘poropuntiinae’ species arranged from Giemsa- stained, Ag-NOR banding chromosomes (arrows) and chromosomes after FISH with 5S (red) and 18S (green) rDNA probes. 1 = *C. harmandi*; 2 = *C. apogon*; 3 = *H. malcolmi*. Chromosomes were counterstained with DAPI (blue). Scale bar = 5 μm.

**Figure 3:**
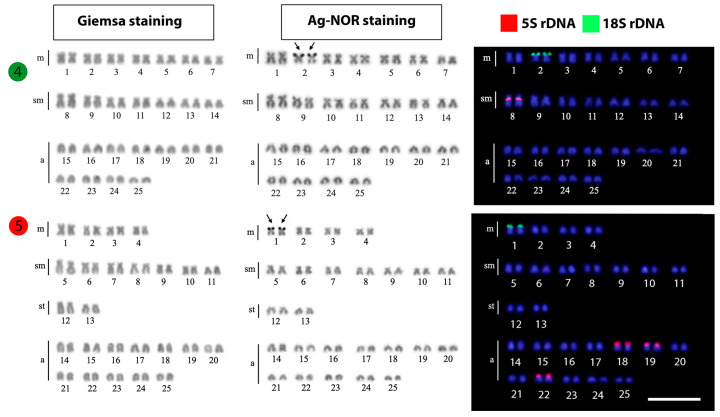
Karyotypes of ‘poropuntiinae’ species arranged from Giemsa-stained, Ag-NOR banding chromosomes and chromosomes (arrows) after FISH with 5S (red) and 18S (green) rDNA probes. 4 = *H. wetmorei*; 5 = *M.* *chilopterus*. Chromosomes were counterstained with DAPI (blue). Scale bar = 5 μm.

**Figure 4:**
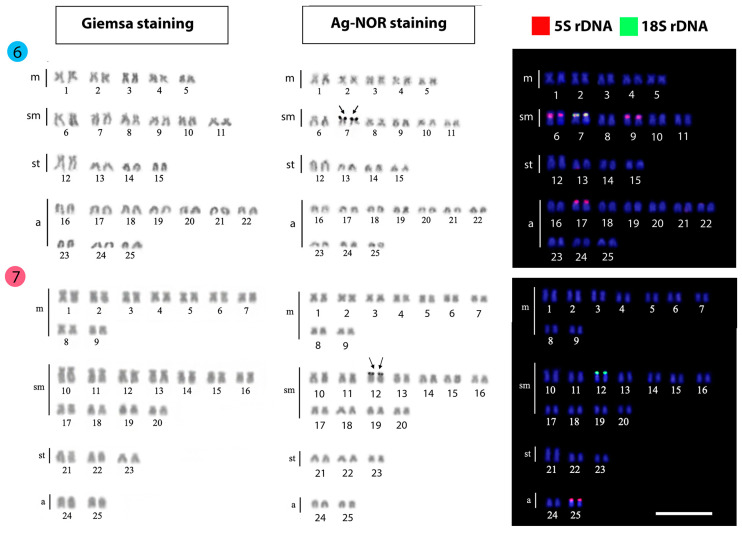
Karyotypes of ‘poropuntiinae’ species arranged from Giemsa-stained, Ag-NOR banding chromosomes (arrows) and chromosomes after FISH with 5S (red) and 18S (green) rDNA probes: 6 = *M. ectypus*; 7 = *P. proctozystron*. Chromosomes were counterstained with DAPI (blue). Scale bar = 5 μm.

All seven studied species had 2n = 50 in both females and males, but different karyotype compositions (Figure 2, Figure 3 and Figure 4 and Table 1). We were unable to detect sex chromosomes in any of the species examined. Ag-NORs were always found near the terminal region of all chromosomes of all species, except for *H. malcolmi*, in which they were located in the pericentromeric area of the first chromosome pair (Figure 2 and Figure 3).

### 3.2. FISH-Mapping

The 18S rDNA probe hybridized to a single chromosome pair in *H. wetmorei*, *M. chilopterus*, *M. ectypus* and *P. proctozystron*. Two chromosome pairs carried these sites in *C. harmandi* and *H. malcolmi*, whereas three pairs were found in *C. apogon*. Except for *H. malcolmi*, which was found in both the centromeric and telomeric regions of the p arms, the 18S rDNA was found in the telomeric region of the p arms (Figure 2 and Figure 3). The distribution of the 5S rDNA site, on the other hand, varied significantly, ranging from one chromosomal pair in *C. harmandi*, *C. apogon*, and *P. proctozystron* to two chromosome pairs in *H. wetmorei* and *H. malcolmi*, three in *M. chilopterus*, and four pairs in *M. ectypus*. Except for *H. malcolmi* and *M. ectypus*, where the 5S rDNA sites were located in both the pericentromeric and telomeric regions, they were present in the telomeric region of the p arms in nearly all species (Figure 2, Figure 3 and Figure 4).

**Figure 5:**
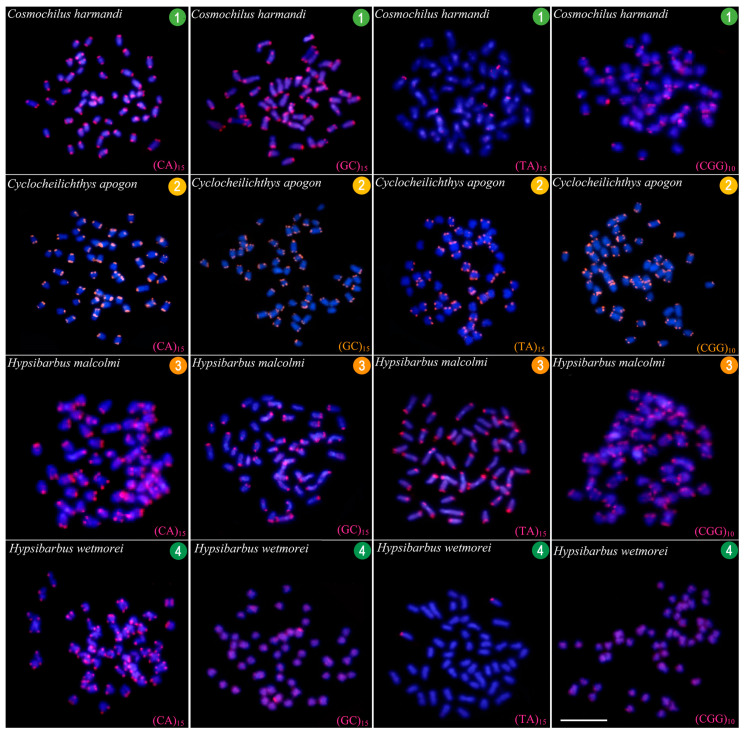
Metaphase plates of *Cosmochilus harmandi*, *Cyclocheilichthys apogon*, *Hypsibarbus malcolmi*, and *Hypsibarbus wetmorei* after FISH with different microsatellite motifs. Scale bar = 5 μm.

**Figure 6:**
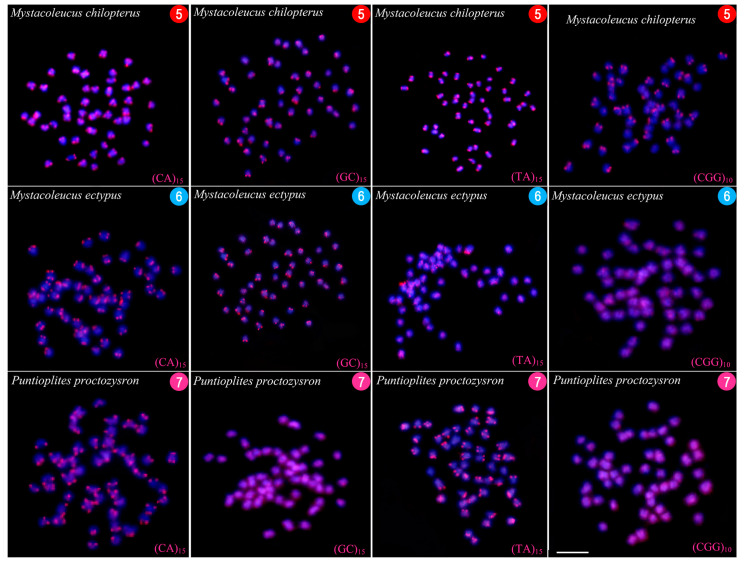
Metaphase plates of *Mystacoleucus chilopterus*, *Mystacoleucus ectypus* and *Puntioplites proctozystron* after FISH with different microsatellite motifs. Scale bar = 5 μm.

The chromosomal mapping of (CA)n revealed the same hybridization pattern in the telomeric regions of many chromosomal pairs across all species. The same situation occurred with (GC)n, but *H. wetmorei* again experienced substantial hybridization in a single pair’s telomeric region. (TA)n followed a similar pattern, being dispersed over all chromosomes but with significant signals in the telomeric region of a single pair in *M. ectypus* and *H. wetmorei*. Furthermore, (CGG)n accumulates in the telomeric regions of all species, in addition to two pairs and in the pericentromeric region of a single chromosomal pair (Figure 5 and Figure 6).

### 3.3. CGH-Studies

**Figure 7:**
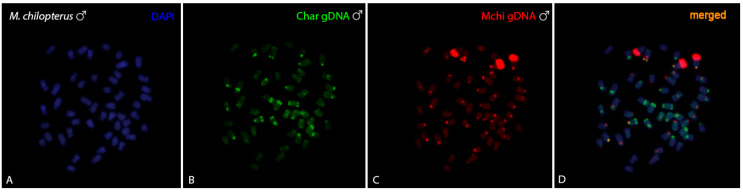
Comparative genomic hybridization (CGH) for interspecific comparisons. Male-derived genomic probes from *Cosmochilus harmandi* (Char—green) and *Mystacoleucus chilopterus* (Mchi—red) hybridized together onto male chromosomes of *M. chilopterus* (**A**–**D**). The common genomic regions are depicted in yellow on the last column (**D**).

The gDNA comparison of *C. harmandi* (Char gDNA, Figure 7B) and *M. chilopterus* (Mchi gDNA, Figure 7C), hybridized in male metaphase chromosomes of *M. chilopterus* (Figure 7A) indicated a high degree of genomic divergence between species, as evidenced by the great number of non-overlapped signals (Figure 7D). The Char gDNA was hybridized to many centromeric areas, the majority of which were shared with the Mchi gDNA. Furthermore, certain chromosomal pairs displayed unique hybridization signals in the telomeric region with Char gDNA, whereas Mchi gDNA presented exclusive sites in centromeres, as well as three chromosomes exhibiting strong hybridization in the telomeric region (Figure 7D).

## 4. Discussion

The family Cyprinidae s.str. (*sensu* [1]) includes 11 lineages, from which 8 altogether contain evolutionarily tetraploid and hexaploid forms beside diploid ones, whereas only 3 include exclusively diploid representatives, namely Acrossocheilinae, Labeoninae, and Poropuntinae. All Poropuntiinae species under investigation, as well as those previously studied (Table 1), have a diploid chromosomal number equal to 2n = 50, confirming their diploid status.

This chromosomal number is also seen in diploid members of other cyprinid lineages, in addition to diploid Acrossocheilinae and Labeoninae [2,3,54]. Indeed, 2n = 50 appears to be preserved in various cyprinid and cobitoid fish lineages [35]. However, such conserved 2n is evidently associated with extensive intrachromosomal variations, which stress the role of structural rearrangements, such as pericentric inversions, chromatin additions/deletions, transpositions, and non-Robertsonian translocations as, e.g., demonstrated by [55] in other cyprinoid lineage, chondrostomine species (Leuciscidae).

**Figure 8:**
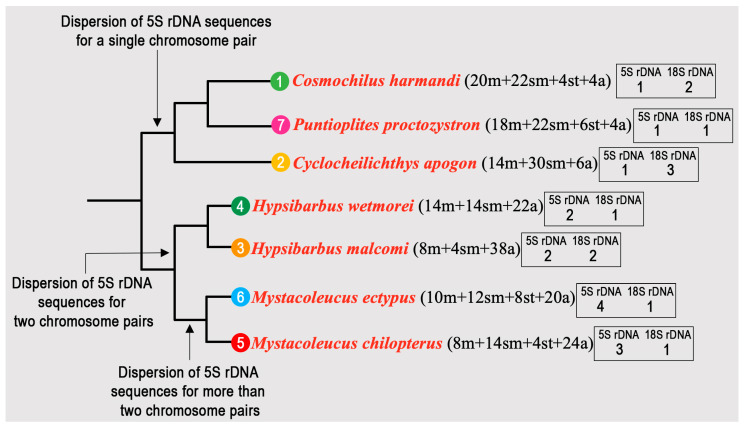
Adapted phylogenetic tree for the tribe Poropuntiinae, based on the molecular-phylogenetic data generated by [2,3], with chromosomal data mapped on it. Superscript numbers correspond, respectively, to the amount of 5S and 18S rDNA loci in their karyotypes.

The Ag-NOR stained regions corresponded to the 18S rDNA loci in all studied species (Figure 2, Figure 3 and Figure 4), except *H. malcomi*, which had an extra site in the pericentromeric region. This means that all 18S rDNA sites in poropuntiins were transcriptionally active, due to the presence of nucleolin and nucleophosmin, two argyrophilic proteins involved in rRNA transcription and processing, and the targets of the Ag-NOR stain approach [56]. The majority of cyprinoid species possess this pattern [33], hypothesized to be the ancestral pattern across cypriniform fishes, but several sites, as shown in *C. harmandi*, *C. apogon*, and *H. malcomi*, were classified as derived ones [35]. In contrast to 5S rDNA, where the number of loci is likely to be associated with the diversification of clades, 18S rDNA distribution pattern does not follow a phylogenetic trend (Figure 8). Therefore, we hypothesized that in comparison with its sister clades, the *Hypsibarbus* + *Mystacoleucus* clade had a greater number of chromosomal rearrangements. The dynamic of ribosomal gene clusters was known to promote large intragenomic diversification [6,57,58,59,60]. The rDNA clusters in all eukaryotes were made up of four units: the 18S rDNA (40S ribosomal subunit), the 5S, 5.8S, and 25-25S (60S ribosomal subunit), and the 5S, 5.8S, and 25-25S (60S ribosomal subunit) (reviewed in [61]). The syntenic arrangement of both 5S and 18S rDNAs, as observed in *H. malcomi*, do not seem to have a functional role on ribosomes and then can be considered a simple result of the intrinsic high dynamic of those sequences [62,63].

In contrast to the loci of 5S rDNA identified in the clade *Hypsibarbus* + *Mystacoleucus*, a single locus of 5S rDNA may indicate a derived trait, according to the phylogenetic reconstruction proposed by [3] (Figure 2 and Figure 3). As a result, we selected a representative from each branch and compared their genomes using CGH to see if they also presented a genomic variation associated with their repetitive DNA content. This genome comparison technique has been applied to several teleost families, including the Salmonidae [64], Characidae [65], Cichlidae [66], Siluridae [67], and cyprinoids with small genome sizes, such as the Iberian Leuciscidae [68] or Carassius [69]. The remarkable chromosomal dynamism in both *C. harmandi* and *M. chilopterus* species corresponded with high dynamics in their repetitive DNA content, evidenced by a variety of non-overlapping signals revealing sequence conservation among their genomes, particularly in centromeric regions (Figure 7). A similar dynamic situation was observed after microsatellite mapping (Figure 5 and Figure 6). Microsatellite motifs are abundant in the heterochromatic regions of fish genomes (telomeres, centromeres, and sex chromosomes) (reviewed in [10]). The genome of *C. harmandi* had hybridizations in the centromeric areas, whereas other species showed signals in the telomeric regions in the previously examined species. The sequences (GC)n, (TA)n, and (CGG)n were found in the terminal region of several chromosomes of *H. wetmorei*, *M. ectypus*, and *C. harmandi* (Figure 5 and Figure 6), respectively. Because repetitive DNAs are abundant in eukaryotic genomes and evolve more quickly, their role as the primary mechanism in inducing karyotype rearrangements has been intensively studied [10].

Karyotypes of cyprinoid fishes usually contain a significantly higher proportion of biarmed chromosomes than uniarmed ones [5,70]. However, the representatives *Hypsibarbus* + *Mystacoleucus* clade had a significantly higher number of acrocentric chromosomes than its sister clade, which includes *Cyclocheilichthys*, *Puntioplites*, and *Cosmochilus* (Figure 8). The same was true for other poropuntiin genera such as *Balantiocheilos*, *Barbonymus*, *Poropuntius*, *Puntioplties*, *Scaphognathops*, and *Sikukia* (Table 1). Other populations of *C. harmandi* [26] exhibit similar characteristics, contradicting the reported trend. However, cypriniform chromosomes are noticeably small, making classification of the exact centromere position difficult and hence the proper assignment of chromosomes into chromosome categories problematic. This might explain why karyotype reports differ between populations and species [6,70,71,72,73]. In the other sister lineage Smiliogastrinae [2,3], the genus *Hampala*, *Puntius*, and *Systomus* also have a high number of acrocentric chromosomes in their karyotypes (reviewed by [16]). Thus, a high number of acrocentric chromosomes might be a plesiomorphic feature of both the Poropuntiini and Smiliogastrini tribes. The presence of karyotypes composed primarily of mono-armed chromosomes (acrocentric) appears to be a feature of most derived fish clades, whereas the basal ones exhibit primarily biarmed ones (meta-, submetacentric) [74]. Aside from the differences found in fish phylogeny between basal and derived orders, the tendency towards chromosome acrocentrization appears to occur even within groups at the family level, as seem in some Neotropical and marine groups [15,75]. Considering the diversity of freshwater fishes, Cypriniformes can only be considered a basal clade when compared to Characiformes, Siluriformes and Gymnotiformes, but not within Acipenseriformes and Osteoglossomorpha [76]. It is important to note that a single karyotypic feature of a particular clade may not represent the entire evolutionary trend of cypriniforms. The high proportion of acrocentric chromosomes in this case could be due to pericentric inversions, which occur when a chromosome segment breaks off, rotates 180 degrees, and reattaches to the same chromosome in the opposite orientation. This inversion can result in the reversal of gene order along the chromosome and is a significant event in the diversification of karyotypes. While karyotypic features can be used to identify relationships between organisms, it is critical to consider all available evidence when determining the evolutionary history of a specific group.

## 5. Conclusions

Our findings have expanded the knowledge of karyotypes and chromosomal characteristics of ‘poropuntiin’ fishes. Its species had a conserved 2n of 50, a large number of acrocentric chromosomes in their karyotypes, and as result NF ranging between 62 and 92, indicating large intra-karyotype differentiation. Overall, these patterns suggest that structural chromosomal rearrangements such as pericentric inversions played an important role in the development of this cyprinid lineage. We also demonstrated that ribosomal DNAs and microsatellites have distinct patterns of accumulation in each species, suggesting a high variability of karyotypes while maintaining a level of 2n.

## Figures and Tables

**Table 2:** The collection sites of the seven ‘poropuntiinae’ species and number of analyzed individuals (n).

Species	Hydrographic Basin	*n*
*Cosmochilus harmandi*	Chao Phraya (site 1)	07♀; 06♂
*Cyclocheilichthys apogon*	Mae Klong (site 2)	08♀; 11♂
*Hypsibarbus malcolmi*	Mekong (site 3)	09♀; 09♂
*Hypsibarbus wetmorei*	Mekong (site 4)	07♀; 05♂
*Mystacoleucus chilopterus*	Mae Klong (site 5)	06♀; 08♂
*Mystacoleucus ectypus*	Mae Klong (site 6)	06♀; 06♂
*Puntioplites proctozysron*	Mae Klong (site 7)	07♀; 06♂

## Data Availability

The data presented in this study are available on request from the corresponding author.
